# P01.18. Triterpene acid containing Viscum album L. extracts mediate apoptosis in paediatric solid cancer cells

**DOI:** 10.1186/1472-6882-12-S1-P18

**Published:** 2012-06-12

**Authors:** G Kauczor, C Delebinski, S Jäger, K Seeger, G Seifert

**Affiliations:** 1 Charité, Department of Pediatric Oncology/Hematology, Berlin, Germany; 2 Betulin-Institute , Darmstadt, Germany

## Purpose

Paediatric solid cancers such as osteosarcoma, Ewing´s sarcoma, rhabdomyosarcoma and neuroblastoma are the most common cancers in children besides leukemia. These cancers have a poor prognosis, are highly metastatic and often resistant to current therapeutic approaches. Viscum album L. (mistletoe) is one of the most widely used complementary cancer therapies in Germany but little is known about its actual effects on paediatric solid cancers. Approved Viscum album L. extracts (VAE) basically contain water soluble compounds of the plant (lectins, viscotoxins). However, mistletoe also contains triterpene acids (mainly oleanolic- and betulinic acid) that are water-insoluble. The anti-tumorigenic properties of these solubilized triterpene acids are the subject of ongoing research. The aim of this study is to determine the effects of different VAE containing either lectins (viscum), triterpene acids (TT) or a combination thereof (viscum TT) on solid tumor models *in vitro* and *in vivo*.

## Methods

Ewing´s-, osteo- and rhabdomyosarcoma as well as neuroblastoma cell lines were treated with the above-mentioned different VAE. The cytotoxicity and the induction of apoptosis were determined using mitochondrial membrane potential measurements (JC-1) and Annexin/PI assays. The mechanism of apoptosis was further analyzed by Western blot analysis and caspase inhibitors. *In vivo* efficacy was determined in the spontaneous NXS2 metastases model.

## Results

First experiments have shown diverse but effective levels of sensitivity for triterpene acid containing mistletoe extracts among the tested tumor cell lines. The JC-1 assays revealed a mitochondria-dependent apoptosis induction. Results on the efficacy of treatment with VAE in neuroblastoma *in vivo* will be presented at the meeting.

## Conclusion

We could demonstrate that Viscum album L. extracts inhibit cell proliferation in different paediatric solid cancers cell lines and in a neuroblastoma model *in vivo*, supporting the effectiveness of these extracts as adjuvant tumor therapy. Furthermore, the results revealed cell-line-specific differences of the apoptosis rate induced by VAE.

